# Fusobacterial liver abscess: a case report and review of the literature

**DOI:** 10.1186/s12879-017-2548-9

**Published:** 2017-06-20

**Authors:** Dilip Jayasimhan, Linus Wu, Paul Huggan

**Affiliations:** 1 Department of Medicine, Waikato Hospital, Level 2 Waiora Waikato Building, Pembroke Street, Hamilton, 3204 New Zealand; 2 Department of General Surgery, Waikato Hospital, Level 2 Waiora Waikato Building, Pembroke Street, Hamilton, 3204 New Zealand

**Keywords:** Fusobacterium, Liver abscess, Review, Case reports, Lemierre’s syndrome, Periodontal diseases

## Abstract

**Background:**

Fusobacteriae are facultative anaerobic gram-negative bacilli which cause a range of invasive infections, amongst which pyogenic liver abscesses are rare. We describe a case of Fusobacterium nucleatum liver abscess and review the relevant literature.

**Case presentation:**

A 51-year-old lady presented with a 4-day history of abdominal pain, diarrhoea, fever, rigors, and lethargy. Imaging revealed an abscess which was drained. Cultures of the blood and abscess aspirate grew *Fusobacterium nucleatum* and *Prevotella pleuritidis* respectively. She achieved full recovery following treatment.

A MEDLINE search was undertaken using free-text and Medical Subject Headings (MeSH), keywords “Fusobacterium” and “Liver abscess”. Non-English language reports and cases without confirmed growth of Fusobacterium species were excluded. Additional cases were identified by surveying the references of each report and by using the same keywords in a web-based search.

Forty-eight cases were identified, 41 in men. The median age was 42.5, with an interquartile range of 33. F. nucleatum and F. necrophorum were in involved in 22 cases each, and 4 cases were not further speciated. Among cases of F. nucleatum liver abscess, nine were attributed to periodontal disease, four to lower gastrointestinal tract disease, one to Lemierre’s Syndrome, and eight were considered cryptogenic. All patients treated made a full recovery. Antimicrobial treatment duration ranged from 2 weeks to 6 months with a median of 6 weeks.

**Conclusion:**

Fusobacterium nucleatum is an uncommon cause of liver abscess generally associated with good clinical outcomes with contemporary medical and surgical care.

## Background

*Fusobacteriae* are arguably unique amongst non-spore forming anaerobes in their ability to cause severe, clinically distinct, mono-microbial infections in a range of organs, often with severe sequelae as originally described by Lemierre and others in the first half of the twentieth century [1]. Fusobacterial infections can affect the head and neck, the lung and pleura, the gastro–intestinal organs, and the female genital tract [2]. Despite their ability to cause severe infections, fusobacterium species are uncommonly identified as pathogens in clinical practice, accounting for less than 1% of anaerobic bacteremias. Regional population-based studies have described the incidence of Fusobacterial bacteraemia to be around 0.55 per 100,000 people per year [3, 4], and that of clinically significant infections to be 0.99 per 100,000 people per year [2]. Whilst the clinical presentations associated with fusobacterial infections are well defined, little is known about the clinical progress and outcomes of specific syndromes [1, 5, 6]. *Fusobacteriae* are a particularly uncommon cause of pyogenic liver abscesses. Therefore, our objective is to describe a case of this condition and to summarise the presentation and outcomes of Fusobacterial liver abscesses through a review of the relevant literature.

## Case presentation

A 51-year-old previously fit and well woman who recently returned from Samoa presented with a four-day history of watery non-bloody diarrhoea, associated with generalised abdominal pain, fevers, chills, and lethargy. Family members reported similar symptoms of milder severity. On admission, she was febrile with a temperature of 38 °C, with otherwise unremarkable vital signs. Examination of the abdomen found a palpable mass in the right upper quadrant. There were no signs of periodontal disease on inspection of the oral cavity. Laboratory tests showed a white cell count of 26.7 × 10E9/L, deranged liver enzymes (alanine transaminase 263 U/L, aspartate transaminase 177 U/L, bilirubin 15 mg/L), and a C-reactive protein of 407 mg/L. A Computed Tomography (CT) scan of the abdomen with contrast revealed a 90 × 92 × 93 mm mixed density lesion in segment II and III of the liver The lesion was percutaneously drained under ultrasound guidance. Cultures of the aspirated abscess fluid and blood both grew anaerobic gram-negative bacilli. Following abscess drainage and treatment with ceftriaxone and metronidazole, the patient continued to experience fevers and diarrhoea and her inflammatory markers did not improve. A follow-up ultrasound scan of the liver revealed an emerging focal abscess in segment IV-b of the liver. The initial CT scan was then retrospectively reviewed to reveal subtle heterogeneous changes in this segment; however, no demarcated fluid collection was present at the time. The two abscesses were not contiguous with but in close proximity to each other. This abscess was also percutaneously drained. Samples from the blood culture were further identified as *Fusobacterium nucleatum* via Biomerieux Vitek (Knowledge database version 2.0) MALDI-TOF (Matrix assisted Laser Desorption/Ionisation - Time of Flight) mass spectrometry with a confidence of 99.9%. Cultures of the initial aspirate were not identifiable using MALDI-TOF and were referred to a quaternary level laboratory in Auckland and later identified as *Prevotella pleuritidis* through 16 s ribosomal Ribonucleic Acid identification. Both specimens underwent susceptibility testing using Clinical Laboratory Standards Institute (CLSI) – recommended media and breakpoint determination using MIC gradient strips. The *Prevotella pleuritidis* was tested on Brucella agar with sheep blood, haemin, and vitamin K, while the *F. nucleatum* required Brain Heart Infusion agar to achieve satisfactory growth.

Given the growth of a second organism common to the periodontal flora and in the absence of symptoms, signs, or imaging evidence of bowel pathology, a periodontal, rather than a gastrointestinal, source was considered more likely and no further evaluation of the large bowel was undertaken.

Treatment was changed on day nine to a two-week course of IV ertapenem, prior to susceptibilities being known, followed by four further weeks of oral amoxicillin-clavulanate. The patient recovered fully.

Although *Fusobacterium* was only grown in the blood culture and not in the aspirate culture, we were confident in treating this case as a *Fusobacterial* pyogenic liver abscess. The aspirate sample was collected afterhours and was not processed until 7 hours later. This delay could have significantly affected our ability to identify this anaerobic organism using standard culture methods, especially in the setting of antimicrobial therapy.

## Review of literature

We conducted a literature review of cases of liver abscess associated with *Fusobacterium* species.

### Methods

A MEDLINE Medical Subject Headings (MeSH®) and a free text search were conducted using the keywords “Fusobacterium” AND “liver abscess”. The search was further limited to case reports in the English language and on human subjects. Cases in which a liver abscess was demonstrated by appropriate imaging techniques or direct visualization, and was associated with a positive culture (blood, abscess fluid, or tissue) for a Fusobacterium species were included. Additional studies were included through reviewing the references of the selected case reports. One additional case was identified when the same keywords were entered into a web-based search engine (Google TM, San Francisco, CA).

### Results

Forty-eight cases were identified using the above search strategy. Table 1 summarises them. Forty-one cases involved males, and seven involved females. The age of the patients ranged from 17 to 78 years with a median of 42.5 years and an interquartile range of 33 years. Twenty-two cases identifie*d F. nucleatum* and 22 cases identified *F. necrophorum* as the aetiological pathogen. In another five cases, the microbe was not identified past *Fusobacterium spp.* 29 of 48 cases involved multiple abscesses as opposed to a single abscess. The most common presumed sources were the periodontal flora (12 cases) and Lemierre’s Syndrome (10 cases), followed by the lower GI tract (six cases), the upper GI tract (four cases), and the cervix (one case). Fifteen cases were considered cryptogenic.

**Table 1:** Summary of case reports identified

	Age	Sex	Organism (specimen cultured)	Treatment	Single or multiple abscess(es)	Presumed source
1 [35]	21	M	*F. nucleatum* (abscess aspirate)	- Drainage of the two largest abscesses (R). - Piperacillin/tazobactam (UTP) - Ertapenem (8 weeks)	Multiple	Oropharynx (PF)
2 [36]	48	M	*F. nucleatum* (abscess aspirate)	- Drainage of abscess (R) - Ceftriaxone and metronidazole (3 days) - IV penicillin and metronidazole (10 weeks)	Multiple	Oropharynx (PF)
3 [37]	23	M	*F. nucleatum* (blood culture and abscess aspirate)	- Drainage of abscess (R) - Piperacillin/tazobactam (UTP) - Ertapenem (6 weeks)	Multiple	Unidentified
4 [38]	58	M	*F. nucleatum* (abscess aspirate)	- Drainage of pelvic abscess (S) - Defunctioning colostomy - Ertapenem (6 weeks) - Low molecular weight heparin (2 weeks)	Multiple	Lower GI tract (diverticulitis)
5 [39]	48	M	*F. nucleatum* (abscess aspirate)	- Drainage of two largest abscesses (R) - Piperacillin/tazobactam (14 days) - Moxifloxacin + oral penicillin (14 days)	Multiple	Unidentified
6 [40]	49	M	*F. nucleatum* (abscess aspirate)	- Drainage of largest hepatic abscess(R) - Laparoscopic cholecystectomy - Laparoscopic appendicectomy - IV ertapenem (UTP)	Multiple	Oropharynx (PF)
7 [41]	59	F	*F. nucleatum, Treponema denticola, Prevotella intermedia, Porphyromonas gingivalis, Bacteroides vulgatus* (tissue culture and abscess apsirate culture)	- None	Multiple	Oropharynx (PF)
8 [28]	59	M	*F. nucleatum* (blood culture)	- Imipenem/cilastatin 500 mg BD (4 days) - Oral clindamycin 600 mg BD (UTP)	Single	Oropharynx (PF)
9 [42]	58	M	*F. nucleatum* (abscess drainage)	- Drainage of hepatic abscess (R) - Oral ciprofloxacin, vancomycin, and gentamicin (UTP) - IV metronidazole (12 days) - Oral metronidazole (3 weeks)	Single	Oropharynx (PF)
10 [43]	24	M	*F. nucleatum* (blood culture)	- IV metronidazole (4 weeks) - Oral metronidazole (2 weeks)	Single	Oropharynx (PLS)
11 [44]	62	M	*F. nucleatum* (abscess aspirate)	- Drainage of abscess(NOS) - IV antibiotics (agent and time period not specified)	Single	Lower GI tract (biopsies)
12 [45]	78	F	*F. nucleatum* (abscess aspirate)	- Drainage of hepatic abscess(R) - Drainage of subphrenic collection - Metronidazole (6 weeks)	Single	Lower GI tract (diverticular disease)
13 [46]	68	M	*F. nucleatum* (blood cultures)	- IV cefotaxime 1 g Q8H and IV metronidazole 500 mg Q8H (24 days) - Oral metronidazole (2 weeks) - Enoxaparin 20 mg daily (UTP)	Single	Unidentified
14 [47]	54	F	*F. nucleatum* (abscess aspirate) *S. viridans* (abscess aspirate) *Actinomyces odontolyticus* (abscess aspirate)	- Drainage of hepatic abscess (R) - Extraction of 11 teeth - IV antibiotics (agent and period of treatment unsepciified)	Multiple	Oropharynx (PF)
15 [47]	69	M	*F. nucleatum* (abscess aspirate)	- Drainage of hepatic abscesses (R) - IV antibiotics (unspecified agent or time period) - Dental surgery	Multiple	Oropharynx (PF)
16 [48]	40	M	*F. nucleatum* (blood culture)	- IV cefuroxime, metronidazole, and erythromycin (14 days) - Oral metronidazole (5 weeks)	Multiple	Unidentified
17 [49]	29	M	*F. nucleatum* (abscess aspirate)	- Drainage of the largest abscess (R) - Metronidazole (10 days, route unspecified) - Open drainage other abscesses (S) - Oral metronidazole (4 weeks)	Multiple	Oropharynx (PF)
18 [50]	24	M	*Actinomyces israeli* (abscess aspirate) *F. nucleatum* (Abscess aspirate)	- Open drainage of abscess (S) - Penicillin G 20 million units (4 weeks) - Oral penicillin (6 months)	Single	Unidentified
19 [51]	70	M	*F. nucleatum* (abscess aspirate)	- Metronidazole (2 weeks, route unspecified) - Oral metronidazole (2 weeks)	Multiple	Unidentified
20 [52]	47	M	*F. nucleatum* (tissue culture) Peptostreptococcus (tissue culture)	- Open resection of all three lesions. (S) - IV penicillin (3 weeks) - Repeat laparotomy 49 days later - PO penicillin (4 months)	Multiple	Unidentified
21 [53]	44	F	*F. nucleatum* (blood culture)	- Drainage of abscess (R) - Imipenem and metronidazole (UTP) - Cefotaxime and metronidazole (13 days) - Ceftriaxone and oral metronidazole (5 weeks)	Single	Unidentified
22 [54]	60	M	*F. nucleatum* (abscess aspirate)	- Piperacillin/tazobactam (3 days) - Ertapenem (1 day) - Moxifloxacin (10 days) - Metronidazole (3 weeks)	Multiple	Lower GI tract (diverticulitis)
23 [55]	28	M	*F. necrophorum* (blood culture)	- Ceftriaxone (6 days) - Meropenem (14 days)	Single	Oropharynx (PLS)
24 [56]	30	M	*F. necrophorum* (blood culture)	- Piperacillin/tazobactam and metronidazole (UTP) - Oral levofloxacin and metronidazole (UTP)	Multiple	Unidentified
25 [57]	48	M	*F. necrophorum* (blood culture)	- Cefotiam (UTP) - Doripenem (19 days) - Oral levofloxacin (UTP)	Multiple	Oropharynx (PLS)
26 [29]	40	M	*F. necrophorum* (tissue culture)	- Drainage of abscess (R) - Sulbactam/cefoperazone (10 days) - Meropenem (9 days) - Oral sawacillin (8 weeks)	Single	Unidentified
27 [58]	25	M	*F. necrophorum* (blood culture)	- Piperacillin/tazobactam (UTP) - IV penicillin (6 weeks)	Single	Oropharynx (PLS)
28 [59]	34	M	*F. necrophorum* (left subphrenic collection)	- Vancomycin and meropenem (UTP) - Tigecyclin and meropenem (UTP) - Tigecyclin (4 weeks)	Multiple	Upper GI tract (HPB)
29 [60]	44	F	*F. necrophorum* (blood culture)	- IV ampicillin/sulbactam and ciprofloxacin (5 days) - IV ampicillin/sulbactam and metronidazole (13 days) - IV ampicillin/sulbactam (15 days) - Oral amoxicillin/clavulanate (1 month)	Single	Cervix
30 [61]	18	M	*F. necrophorum* (blood culture)	- Aspiration of abscess (R) - Metronidazole and cefuroxime (UTP) - Piperacillin/tazobactam + clindamycin (5 weeks)	Single	Oropharynx (PLS)
31 [62]	21	M	*F. necrophorum* (abscess drainage) *F. nucleatum* (peritonsillar abscess)	- Drainage of the largest hepatic abscess (R) - Piperacillin/tazobactam and metronidazole (6 weeks)	Multiple	Oropharynx (PLS)
32 [10]	25	M	*F. necrophorum* (abscess aspirate) *Prevotella spp.* (blood culture and abscess aspirate)	- Drainage of abscess - Removal of diseased teeth - Oral amoxicillin/clavulanate (3 weeks)	SIngle	Oropharynx (PF)
33 [11]	64	M	*F. necrophorum* (abscess aspirate)	- Aspiration of abscess (R) - Cefuroxime and metronidazole (2 weeks) - Oral metronidazole and oral ciprofloxacin (UTP)	Multiple	Lower GI tract (haemorrohoids)
34 [63]	22	M	*F. necrophorum* (abscess aspirate)	- Aspiration of abscess (R) - Gentamicin, clarithormycin, metronidazole, ceftriaxone and low molecular weight heparin (6 weeks)	Multiple	Oropharynx (PLS)
35 [64]	19	F	*F. necrophorum* (blood culture and abscess aspirate)	- Drainage of the largest hepatic abscess - IV benzylpenicillin, metronidazole, and ciprofloxacin (5 weeks)	Multiple	Oropharynx (PLS)
36 [65]	71	M	*F. necrophorum* (abscess aspirate)	- IV benzylpenicillin (6 weeks) - Oral amoxicillin (6 weeks)	Multiple	Lower GI (diverticular disease)
37 [66]	44	M	*F. necrophorum* (abscess aspirate) H. Parainfluenzae (abscess aspirate)	- Drainage of hepatic abscess (R) - IV tetracycline (3 days) - Co-trimoxazole (8 days) - IV cefazolin + IV gentamicin (UTP) - IV ceftriaxone (3 weeks)	Single	Unidentified
38 [67]	18	M	*F. necrophorum* (blood culture)	- Cefotaxime, vancomycin, and azithromycin. Then cefepime and metronidazole (unspecified chronology or time period)	Single	Oropharynx (PLS)
39 [68]	17	F	*F. necrophorum* (blood culture)	- Nafcillin and ceftriaxone (3 days) - Nafcillin, ceftriaxone, and metronidazole (6 weeks)	Multiple	Oropharynx (PLS)
40 [69]	27	M	*F. necrophorum* (blood cultures and abscess aspirate)	- Drainage of hepatic abscess (R) - IV ampicillin, gentamicin, and metornidazole (10 days) - Oral ampicillin and metronidazole (5 weeks)	Single	Oropharynx (PLS)
41 [70]	55	M	*F. necrophorum* (abscess aspirate)	- Aspiration of abscess - IV ampicillin/sulbactam (1 month) - Oral amoxicillin/clavulanate (21 days)	Multiple	Upper GI tract (HPB)
42 [71]	31	M	*F. necrophorum* (abscess aspirate)	- Open drainage of abscesses (S) - Gentamicin, clindamycin, and doxicycline (15 days) - Further open drainage of abscess (S) - IV chloremphenicol and metronidazole (3 weeks)	Multiple	Unidentified
43 [72]	36	M	*F. necrophorum* (abscess aspirate and blood culture)	- Drainage of hepatic abscesses (R) - Ceftriaxone 2 g Q24H (UTP) - Ampicillin/sulbactam 12 g/day (UTP)	Multiple	Oropharynx (PF)
44 [73]	57	M	*F. necrophorum* (abscess aspirate)	- Drainage of abscess (R) - Metronidazole (7 days) and ceftriaxone (UTP) - Piperacillin/tazobactam (4 weeks)	Single	Oropharynx (PF)
45 [74]	34	M	*Fusobacterium spp.* (abscess aspirate)	- Drainage of largest abscess with gentamicin flushes. (R) - Drainage of subdiaphragmatic collection (R) - Aspiration of smaller abscesses. (R) - Imipenem (22 days)	Multiple	Upper GI tract (Crohn’s Disease)
46 [16]	69	M	*Fusobacterium spp*. (liver biopsy)	- Piperacillin and metronidazole (3 weeks) - Oral amoxicillin/clavulanate and metronidazole (2 months)	Multiple	Unidentified
47 [75]	41	M	*Fusobacterium spp.* (abscess aspirate)	- Drainage of hepatic abscess (R) - Cefomandole, gentamicin, and metronidazole (UTP) - Penicillin (1 month)	Single	Upper GI tract (diverticulitis of terminal Ileum)
48 [76]	22	M	*Fusobacterium Spp* (blood cultures)	- Penicillin (31 days)	Multiple	Unidentified

*PLS* Pharyngitis/Lemierre’s syndrome, *PF* Periodontal flora, *UTP* Unspecified time period, *IV* Intravenous, *BD* twice daily, *R* radiological drainage, *S* surgical drainage

Among the cases that identified *F. nucleatum*, the most common presumed source was the periodontal flora (nine out of 22 cases). Eight cases were cryptogenic. The presumed source was considered to be the lower GI tract in four cases, and Lemierre’s syndrome in one case.

Treatment mostly consisted of abscess drainage and prolonged antimicrobial therapy. The duration of treatment was specified in 39 cases. Among them, the minimum duration was 2 weeks, and the maximum was 6 months, with a median of 6 weeks, and an interquartile range of 2 weeks. The duration was less than 4 weeks in five cases, between four to 8 weeks in 26 cases, and more than 8 weeks in eight case. Commonly used antibiotics include beta-lactams (in combination with metronidazole in some cases), carbapenems, metronidazole monotherapy, and less frequently fluoroquinolones.

Treatment outcomes were favourable with 47 of 48 cases reporting complete recovery.

## Discussion

Whilst the essential features of disease caused by *Fusobacteriae* have been recognised for over a century, presentations remain uncommon and widely distributed across medical specialties [1]. Thus the historical background, aetiopathogenesis, and likely clinical outcomes of fusobacterial disease are poorly understood by most clinicians. This report demonstrates the common features of fusobacterial liver abscess and by combining individual reports and case series provides insights into the associated spectrum of underlying illness.

Loeffler identified a thin, gram-negative rod present in necrotic tissue in calf diptheriae in 1884 [7]. The term ‘necrobacillosis’ was coined by Bang, who isolated the organism from diseased cattle and pigs [8]. Koch’s postulates were satisfied accidentally when Schmorl and an assistant suffered pyogenic infection of their fingers whilst working with diseased rabbits, samples from both of which yielded *Fusobacteriae* [5]. Cases of de novo necrobacillosis were described in humans by the turn of the twentieth century. Lemierre gave his classic dissertation on anaerobic bacteremia in 1936, earning the right to an eponymous syndrome of tonsillar abscess progressing to internal jugular vein thrombosis, distant abscess formation, and overwhelming sepsis [9].

In the twentieth century it was axiomatic, based on very little evidence, that *Fusobacteriae* were part of the normal human oropharyngeal, gastro-intestinal (GI) tract and female genital tract flora. The apparent rarity of illness caused by these organisms reinforced the notion that they were opportunists, causing illness only when natural barriers to infection were compromised [1, 10, 11]. Contemporary studies have shed light on the true environmental niche and pathogenic potential of *Fusobacteriae* in general and *Fusobacterium nucleatum* specifically. *Fusobacterium nucleatum* is a major constituent of the gingival, oropharyngeal and together with *F. necrophorum*, the appendiceal flora. Neither organism is a component of the healthy colonic flora nor do they form a major component of the healthy vaginal micro-biome [1, 12, 13]. *F. necrophorum* was found to exist in the healthy oropharyngeal flora in only one polymerase chain reaction (PCR) based study reported and in none of the culture-based studies [1]. Gingivo-periodontitis, endodontic infection and suppurative appendicitis occur in the setting of clonal expansion and invasion of *Fusobacteriae,* and it has been postulated that adverse obstetric outcomes arise from haematogenous spread of *F. nucleatum* to placental tissue [14, 15]. *Prevotella pleuritidis* was identified in the case we described and the gingival/oropharyngeal flora were identified as co-pathogens in 18% of liver abscess caused by *F. nucleatum* in our review of the literature.

Indeed, our review of fusobacterial pyogenic liver abscesses showed a low prevalence of general risk factors for liver abscess including malignancy, dialysis treatment, and older age [4]. Most cases occurred in young or middle-aged, immunocompetent individuals with risk factors for haematogenous spread of *Fusobacteriae* such as periodontal disease and recent pharyngitis.

Attribution of the lower GI tract as a source of liver abscess was relatively uncommon and the grounds for doing so were often unclear. The utility of investigating for a presumed source is unknown at this point due to the lack of research assessing this particular question. Our findings support the contention that the majority of disseminated fusobacterial infections originate from the oral cavity even without symptoms of a dental disease. Dental X-Rays to identify possible dental abscesses and cavities or more sophisticated imaging modalities of the oral cavity and the sinuses may be of the most benefit, should clinicians choose to investigate the underlying sources of bacteremia in patients without localising symptoms [16].

Among cases where F. necrophorum were cultured, only three of the 23 liver abscesses were associated with a co-pathogen. None of these cases had clinical findings consistent with classic Lemierre syndrome. Whilst the environmental niche of *F. necrophorum* is less understood than that of *F. nucleatum,* it is among the most commonly identified cause of tonsillar abscess after Lancefield Group A beta-hemolytic streptococci [17, 18]. This potentially points to a more evolved role for *F. necrophorum* as a human pathogen.

We observed good outcomes with appropriate source control and antimicrobial therapy. The only case involving a fatality was a post-mortem diagnosis and treatment had not been initiated. There was significant variation between studies with regards to the choice and duration of antimicrobial therapy. Current evidence suggests the need for prolonged antimicrobial therapy for patients with liver abscess and risk factors for treatment failure. These include malignancy, older age, presence of septic shock at presentation, certain biochemical derangements (e.g. anaemia, azotaemia, and hyperbilirubinaemia), and an Acute Physiology and Chronic Health Evaluation (APACHE) II score of more than 15 [19–21].

Routine prolonged broad-spectrum antimicrobial therapy may not be warranted in cases of fusobacterial liver abscess with a presumed oral or periodontal origin, especially in the setting of abscess drainage. *Fusobacteriae* remain highly susceptible to beta-lactams. Studies on susceptibility patterns have shown no increase in amoxicillin-resistant *Fusobacteriae* over the past decade [22–24]. They are unlikely to be accompanied by highly resistant co-pathogens in polymicrobial infection of oral or periodontal origin. Metronidazole has been associated with good clinical responses in the treatment of fusobacterial and mixed anaerobic infections [25]. Where source control is achieved, and with the aid of reliable clinical biomarkers such as C-reactive protein, antimicrobial therapy may be shortened [26, 27]. Further research into shorter course therapy for highly sensitive organisms such as *Fusobacteriae* could better delineate optimal antimicrobial treatment duration for patients with liver abscesses.

The strengths of this review are that it employed an extensive search strategy to identify a greater number of cases than previous reviews on the same topic. Previous literature reviews of case reports have focused mainly on only one *Fusobacteriae* species (*F. necrophorum* or *F. nucleatum*) [28, 29]. To our knowledge, this is the first review to include all *Fusobacteriae spp.,* to demonstrate their homogeneity in clinical presentation, clinical findings, co-pathogens and outcomes.

Our study has several weaknesses*.* The main weakness is that in the case presented, *Fusobacterium nucleatum* was identified in the blood culture but not in the abscess aspirate. We remain confident in attributing *F.nucleatum* as a causative co-pathogen for a number of reasons. Firstly, delays in sample processing have been known to increase the difficulty in identifying anaerobes through conventional culture methods [30], especially in the setting of antimicrobial therapy, which was commenced in advance of abscess drainage. Studies using pyrosequencing of direct samples have shown that pyogenic abscesses are frequently polymicrobial, with the number of aetiologic agents identified significantly exceeding what is achieved using standard culture-based methods [30, 31]. Secondly, many population-based studies accept blood cultures as an appropriate standard in diagnosing causative organisms and in guiding antimicrobial treatment in pyogenic liver abscess [32]. Some cases included in our review identified *Fusobacteriae* by blood culture alone [33, 34]. Therefore, we have grounds to believe that *F. nucleatum* was indeed a causative agent.

In terms of other weaknesses, we reported only published observations; publications may have been biased towards those of specific interest to a journal’s readership or those that were associated with good clinical outcomes. Cases in languages other than English were excluded. Attribution of underlying source was presumptive rather than definitive in a significant number of case series. Furthermore, there was minimal information on microbiologic methods. This may have led to an over-reporting of mono-microbial infection, for example, where culture-based methods lacked sensitivity or where growth of the anaerobic flora at low quantities was under-reported.

## Conclusion

In this case report and review of the literature, we found that fusobacterial liver abscesses resulted in good outcomes with contemporary medical and surgical care.

## Abbreviations

BD: Twice daily
CLSI: Clinical Laboratory Standards Institute
CT: Computed tomography
DNA: Deoxyribonucleic acid
GI: Gastrointestinal
IV: Intravenous
L: Litre
LS: Lemierre’s syndrome
mg: Milligrams
mm: Millimetre
PCR: Polymerase chain reaction
PF: Periodontal flora
R: Radiological drainage
S: Surgical drainage
U: Units
UTP: Unspecified time period
